# Parental awareness and dental health behavior of children with congenital heart disease, with diabetes mellitus, or undergoing anti-cancer treatment, compared to healthy children

**DOI:** 10.3389/froh.2024.1435070

**Published:** 2024-10-25

**Authors:** Elinor Halperson, Hanan Badarneh, Ella Zion, Helly Kruchenezki, Gal Goldstein, Sagui Gavri, David Zangen, Avia Fux-Noy

**Affiliations:** ^1^ Department of Pediatric Dentistry, Hadassah Medical Center, Faculty of Dental Medicine, Hebrew University of Jerusalem, Jerusalem, Israel; ^2^ Faculty of Dental Medicine, Hebrew University of Jerusalem, Jerusalem, Israel; ^3^ Department of Pediatric Hematology-Oncology, Hadassah Medical Center, Faculty of Medicine, Hebrew University of Jerusalem, Jerusalem, Israel; ^4^ Department of Pediatric Cardiology, Hadassah Medical Center, Faculty of Medicine, Hebrew University of Jerusalem, Jerusalem, Israel; ^5^ Department of Pediatric Endocrinology, Hadassah Medical Center, Faculty of Medicine, Hebrew University of Jerusalem, Jerusalem, Israel

**Keywords:** dental health, oral health, children, awareness, systemic diseases

## Abstract

**Introduction:**

Children with chronic diseases tend to experience a considerably higher burden of oral disease compared to their healthy peers. Low awareness of the impact of systemic diseases on oral health, lack of motivation, and discomfort may render the maintenance of good oral hygiene challenging. We conducted a study of four groups of parents: of children with congenital heart disease (CHD), with diabetes mellitus type 1 (DM), and undergoing anti-cancer treatment (ACT); and a control group of healthy children (C). We aimed to compare between the groups, parental attitudes and knowledge of their children's oral health, and their reports of their children's dental habits.

**Methods:**

Parents who arrived with their children for routine check-ups at three main clinics: cardiology, endocrinology, and hematology-oncology were asked to respond to a questionnaire regarding their children's oral and dental health.

**Results:**

A total of 287 questionnaires were collected from 76 parents of children with CHD, 100 parents of children with DM, 50 parents of children undergoing ACT, and 61 parents who comprised group C. Compared to the CHD and DM groups, the ACT group demonstrated significantly more awareness of the importance of maintaining oral and dental health following diagnosis of their children's medical condition. Mothers’ education was found to correlate with dental health knowledge. Most children in the DM and C groups had previous dental examinations, compared to only half in the CHD and ACT groups. A higher proportion of the children in the C than the other groups brushed teeth twice daily. The groups were similar in their consumption of sugary drinks, and of sweets and snacks. The children's specialist physicians were the main source of information on oral health, especially in the ACT group.

**Conclusions:**

Although most of the parents reported awareness to the oral health aspects of their children's disease, only part of them reported that their children visited dentists, and brushed their teeth twice daily. For the parents of children with CHD, DM, and ACT, their children's treating specialists were the primary source of information regarding oral health. This highlights the importance of clear and continuous communication between pediatric specialists and dentists.

## Introduction

Compared to their healthy counterparts, children with chronic systemic diseases may be more susceptible to various oral health conditions that require particular health care services (1, 2). These conditions include increased gingivitis, periodontal disease, dental caries, dental crowding, malocclusion, bruxism and wear facets, fracture of teeth or trauma, oral aversion, and anomalies in tooth development, size, shape, eruption, and arch formation (3, 4). When children and their parents cope with a life-threatening disease, less attention may be given to oral and dental health. Low awareness of the effect of the systemic disease on oral health, low motivation, and discomfort may also challenge children's oral health maintenance (4–7). Oral diseases can have a direct and devastating impact on general health and quality of life. Individuals with certain systemic health problems or conditions such as compromised immunity or cardiac conditions may be especially vulnerable to the effects of oral diseases (3). Among random mothers in Tehran, Iran, positive attitudes and knowledge of oral health were associated with their children brushing twice daily and maintaining better oral health (8). This study aimed to assess attitudes, knowledge, and reported behavior regarding dental and oral health, of parents of children with congenital heart disease (CHD), diabetes mellitus type 1 (DM), and undergoing anti-cancer treatment (ACT), compared to healthy children.

## Materials and methods

This cross-sectional descriptive study was based on a convenience sample of parents who arrived with a child aged 6 months to 18 years for a routine check-up at Hadassah Medical Center, Jerusalem. The study involved distributing questionnaires to parents of four specific groups of children: with CHD, with DM, undergoing ACT, and healthy. These three diseases were chosen because they are chronic, life-threatening conditions requiring treatment, hospitalizations, and continuous long-term follow-up in the hospital setting, which may impact their oral health. On the other hand, poor oral health can worsen and put those three groups of children at risk of dental infection and serious oral side effects more frequently than healthy children (control group C). The researchers filled out the questionnaires with the children's parents at the hospital while they waited for a follow-up examination in the relevant department for the child's illness. They asked the parents 14 questions in their native language. Questionnaire accessed information regarding children's oral health and dental habits. It was based on similar questionnaires developed and published by Gupta et al. (9) and Koerdt et al. (10), and was translated by the investigators into Hebrew and Arabic. The questionnaire consisted of five demographic questions, two questions about the child's specific medical condition, and seven additional questions. Two of the latter referred to the parent's awareness of the influence of their child's systemic disease on oral health (q8 and q9), two concerned dental home (q10 and q11. one was about oral hygiene habits (q12), and two were about dietary habits (q13–14). The questions and the answers are detailed in Table 1.

**Table 1:** Parental awareness and dental health behavior questionnaire.

q#					
1	Child's gender	Male	Female		
2	Child's age				
3	Questionnaire respondents	Father	Mother		
4	Fathers’ education	Elementary	High-school	Academic	
5	Mothers’ education	Elementary	High-school	Academic	
6	Child medical condition				
7	Age of the child at the time of medical diagnosis				
8	Do you know the importance of maintaining oral and dental health following your child's medical condition?	YES	NO		
9	Who provided you with the information and explanations regarding the importance of maintaining oral and dental health according to your child's medical condition?	Dentist	Pediatrician	Specialist physician	No one
10	Has your child ever had a dental examination?	YES	NO		
11	How often does your child have a dental examination?	Twice a year or more	Once a year	Less than once a year	
12	How often does your child brush teeth?	Twice a day	Once a day	Less than once a day	
13	How often does your child drink sugary drinks?	Once a day or less	Twice a day or more		
14	How often does your child eat sweets and snacks?	Once a day or less	Twice a day or more		

### Ethics

The study was performed in line with the principles of the Declaration of Helsinki. Approval was granted by the ethical committee on research involving human subjects (0720-20-HMO, 0509-19-HMO, and 0760-21-HMO).

### Statistical analysis

All the data were inserted into Excel software and calculations were performed using SPSS statistical software (version 28.0.; SPSS, Inc., Chicago, Illinois, USA). Quantitative variables were described as means and standard deviations, and as medians and ranges. Qualitative variables were presented by frequencies and percentages. The ANOVA test was used to compare quantitative variables, and the Kruskal-Wallis or Mann-Whitney test to compare variables not normally distributed. Categorical data were compared by using the chi test or Fisher's test. We used logistic regression to analyze the relationship between parents’ awareness of their child's systemic disease and his oral and dental health. All the statistical tests were analyzed at a significance level of *p*-value < 0.05.

## Results

A total of 287 questionnaires were collected, from 76 parents of children with CHD, 100 parents of children with DM, 50 parents of children undergoing ACT, and 61 parents of the C group. Demographic variables are presented in Table 2. The study groups differed in children's age (<0.001); the CHD group was the youngest. The proportions of male and female children were relatively equal, and similar between the groups. In all the groups, most of the respondents to the questionnaire were mothers; 49.5% of the mothers and 40.1% of the fathers had academic educations.

**Table 2:** Demographic variables according to the study groups.

	CHD *N* = 76	DM *N* = 100	ACT *N* = 50	Control *N* = 61	Total *N* = 287	*p*-value
Children's gender *n* (%)						0.461^a^
Male	45 (59.2)	50 (50.5)	23 (46.0)	30 (49.2)	148 (51.5)	
Fema	31 (40.8)	49 (49.5)	27 (54.0)	31 (50.8)	138 (48.5)	
Questionnaire respondents *n* (%)						<0.001^b^
Father	17 (22.7)	30 (30.0)	12 (24.0)	11 (18.0)	70 (24.4)	
Mother	37 (49.3)	70 (70.0)	38 (76.0)	50 (82.0)	195 (67.9)	
Both together	21 (28.0)	0 (0.0)	0 (0.0)	0 (0.0)	21 (7.3)	
Fathers’ education *n* (%)						0.004^b^
Elementary	4 (5.3)	6 (6.0)	8 (16.0)	1 (1.6)	19 (6.6)	
High-school	41 (54.7)	62 (62.0)	25 (50.0)	24 (39.3)	152 (52.9)	
Academic	30 (40.0)	42 (42.0)	17 (34.0)	36 (59.0)	115 (40.1)	
Mothers’ education *n* (%)						<0.001^b^
Elementary	1 (1.3)	6 (6.0)	7 (14.0)	0 (0.0)	14 (4.9)	
High-school	38 (50.0)	58 (58.0)	12 (24.0)	23 (37.7)	131 (45.6)	
Academic	37 (48.7)	36 (36.0)	31 (62.0)	38 (62.3)	142 (49.5)	
Children's age (years)						<0.001^c^
Range	1–18	3.5–15.5	0.8–18	1.8–18	0.8–18	
Median	3.0	10.0	6.6	11.5	9.0	
Mean (SD)	5.0 (4.5)	10.1 (3.1)	8.1 (4.9)	11.2 (3.5)	8.65 (9.0)	

^a^ Pearson Chi-Square.

^b^ Fisher-Freeman-Halton exact test.

^c^ ANOVA.

CHD, congenital heart disease; DM, diabetes mellitus type 1; ACT, ant-cancer treatment; SD, standard deviation.

Differences were found between the groups in dental health awareness, as evident from responses to the third part of the questionnaire (Table 3). Compared to parents in the CHD and the DM groups, those in the ACT group were significantly more aware of the importance of maintaining oral and dental health following their children's medical condition (*p* < 0.001) (q8). The main source of information cited for dental information was children's specialist physicians, especially in the ACT group (*p* < 0.001)(q9). For the CHD group, the self-report of having knowledge was found to correlate with being familiar with the term endocarditis (*p* < 0.001) (Table 4). Sixty-one percent of the parents of child who needed antibiotic prophylaxis were not aware of this need.

**Table 3:** Oral and dental health awareness and habits according to the study groups.

Item from the questionnaire	CHD	DM	ACT	C	Total	*p*-value
Do you know the importance of maintaining oral and dental health following your child's medical condition?						
Yes	33 (43.4)	63 (63.0)	45 (90.0)	NA	141 (62.4)	<0.001^a^
Who provided you with the information and explanations regarding the importance of maintaining oral and dental health according to your child's medical condition?						
Dentist	12 (15.8)	7 (7.0)	11 (22.0)	NA	30 (13.3)	<0.001^b^
Pediatrician	5 (6.6)	5 (5.0)	1 (2.0)	NA	59 (26.1)	
Specialist physician	14 (18.4)	20 (20.0)	25 (50.0)	NA	11 (4.9)	
Has your child ever had a dental examination?						
Yes	38 (50.7)	88 (88.0)	30 (60.0)	58 (95.1)	214 (74.6)	<0.001^a^
How often does your child have a dental examination?						
Twice a year or more	9 (11.8)	12 (12.0)	13 (26.0)	21 (34.4)	55 (19.2)	<0.001^a^
How often does your child brush teeth?						
Twice a day	20 (26.3)	19 (19.0)	19 (38.0)	33 (54.1)	196 (68.3)	<0.001^a^
How often does your child drink sugary drinks?						
Once a day or less	72 (94.7)	90 (90.0)	43 (86.0)	52 (85.2)	257 (89.5)	0.252^a^
Twice a day or more	4 (5.3)	10 (10.0)	7 (14.0)	9 (14.8)	30 (10.5)	
How often does your child eat sweets and snacks						
Once a day or less	68 (89.5)	91 (91.0)	41 (82.0)	48 (78.7)	248 (86.4)	0.097^a^
Twice a day or more	8 (10.5)	9 (9.0)	9 (18.0)	13 (21.3)	39 (13.6)	

^a^ Pearson Chi-Square.

^b^ Fisher-Freeman-Halton exact test.

The data are presented as *n* (%).

CHD, congenital heart disease; DM, diabetes mellitus type 1; ACT, ant-cancer treatment; NA, not applicable.

**Table 4:** Parental awareness of dental health issues following a child's diagnosis with congenital heart disease (*N* = 76).

		Knows the importance of maintaining oral and dental health following diagnose with CHD		Total	*p*-value
		Yes	No		
Familiar with the term “Endocarditis"	Yes	18	1	19	<0.001^a^
	%	54.5%	2.3%	25.0%	
	No	15	42	57	
	%	45.5%	97.7%	75.0%	
Total	n	33	43	76	
	%	100.0%	100.0%	100.0%	
		The child needs AP, according to the cardiologist			
		Yes	No		
The child needs AP- according to the parent	Yes	9	2	11	<0.001^b^
	%	39.1%	3.8%	14.5%	
	No	8	26	34	
	%	34.8%	49.1%	44.7%	
	Does not know	6	25	31	
	%	26.1%	47.2%	40.8%	
Total	n	23	53	76	
	%	100.0%	100.0%	100.0%	

^a^ Pearson Chi-Square.

^b^ Fisher-Freeman-Halton Exact Test.

CHD, congenital heart disease; AP, antibiotic prophylaxis.

The groups differed in their dental home establishment (Table 3). The majority of the children of the DM and C groups had undergone previous dental examinations, compared to only half in the CHD and ACT groups (*p* < 0.001). Lower proportions of the children of the CHD, DM, and ACT groups than of the C group underwent dental check-ups twice yearly: 11.8%, 12.0%, 26.0%, and 34.4%, respectively (*p* < 0.001). Similarly, lower proportions of the children in the three chronic disease groups than in the C group were reported to brush teeth twice daily: 26.3%, 19.0%, 38.0%, and 54.1%, respectively (*p* < 0.001). The parents in the four groups reported similar consumption, by their children, of sugary drinks, and of sweets and snacks (Table 3).

In a multivariate logistic regression model (Table 5), parents in the CHD and DM groups had 15.2 and 5.7, respectively, more chance than the ACT group, of lacking knowledge regarding maintaining oral and dental health. Mothers with an elementary education and mothers with a high school education had 6.8 and 2.1, respectively, more chance to lack knowledge than did mothers with an academic education.

**Table 5:** Results of a multivariate logistic regression model for predicting parental knowledge regarding the importance of maintaining oral and dental health following the diagnosis of a child's medical condition.

		B	S.E	Wald	df	Sig.	Adjusted OR	95% C.I. for OR	
								Lower	Upper
Step 1^a^	Study Group (ACT)			23.159	2	<.001			
	Study Group (CHD)	2.462	.525	21.978	1	<.001	11.727	4.190	32.825
	Study Group (DM)	1.665	.515	10.456	1	.001	5.286	1.927	14.501
	Constant	−2.197	.471	21.725	1	<.001	.111		
Step 2^b^	Study Group (ACT)			24.146	2	<.001			
	Study Group (CHD)	2.724	.585	21.662	1	<.001	15.236	4.839	47.975
	Study Group (DM)	1.740	.568	9.387	1	.002	5.696	1.872	17.335
	Mother's education			10.584	2	.005			
	Mother's education (elementary)	1.921	.688	7.784	1	.005	6.825	1.771	26.307
	Mother's education (high school)	.752	.321	5.494	1	.019	2.121	1.131	3.979
	Constant	−2.847	.558	26.035	1	<.001	.058		

CHD, congenital heart disease; DM, diabetes mellitus type 1; ACT, ant-cancer treatment; S.E., standard error.

^a^ Variable(s) entered on step 1: Study Group.

^b^ Variable(s) entered on step 2: Education mother.

## Discussion

This study focused on three chronic medical conditions in children. Children with CHD have an increased risk of caries due to enamel defects such as hypomineralization, and due to medicines that include a sweetened solution of sucrose. Oral health is often neglected because of long periods of hospitalization at a young age. Some patients take anticoagulant drugs, which increase the risk of bleeding during dental treatment. In addition, transient bacteremia associated with dental procedures is considered a risk factor in the pathogenesis of infective endocarditis, which is a major and serious complication of CHD (6, 11–13). Children with DM type 1 are at increased risk of caries, periodontal diseases, and bacterial infections. Disorders in saliva secretion impair protection of the integrity of the soft tissues in the oral cavity against bacteria, fungi, and viral infections. Children with DM have significantly greater periodontal problems than do healthy children. These include plaque accumulation, calculus, bleeding on probing, and gingivitis (14–16). Children who undergo ACT including immunosuppressive oncology treatments such as chemotherapy, radiotherapy, and bone marrow transplants are at risk of developing acute and long-term oral and dental complications. Acute oral effects may include mucositis, bleeding, taste alterations, secondary infections, salivary gland dysfunction, periodontal conditions, trismus, osteoradionecrosis, and oral neurotoxicity. Late oral phenomena include exacerbated dental caries, temporomandibular dysfunction, osteoradionecrosis, dental developmental anomalies, and oral graft vs. host disease (4, 17).

As CHD is congenital, it was expected that the groups would differ in age. The age difference can also explain differences regarding previous dental examinations; only 50% of the children in the CHD group had ever had one. Providing early dental home to infants and their families is an opportunity to educate parents about their children's oral health. The American Academy of Pediatric Dentistry, the American Dental Association, the European Academy of Paediatric Dentistry, and the International Association of Paediatric Dentistry recommend an oral examination for infants, including newborns, even in the absence of any known problems. A child's first dental visit and oral examination should occur by at least 1 year of age (18–21).

Our findings support previous reports that the higher the education of parents, especially mothers, the greater the likelihood that they would be aware of the importance of maintaining their children's oral and dental health (22, 23). We report that the parents of children receiving ACT, compared to parents of children with CHD and DM, were significantly more aware of the importance of maintaining oral and dental health according to their children's medical condition. Moreover, the dental home establishment and tooth brushing frequency were better among the children with ACT than CHD and DM. These results can be explained by the close cooperation of the Pediatric Dentistry Department and the Hematology-Oncology Department at Hadassah Medical Center. Every child diagnosed with cancer is referred to a dental examination, and treatment if necessary.

Among the three groups of parents of children with a background illness, the main source for understanding the connection between the illness and oral health was the specialist treating the child. It is therefore important that specialist pediatricians will have the relevant information and refer patients for clear continuation and receipt of instructions from dentists, soon after diagnosis of the disease.

Only 43% of the parents of children with CHD reported oral-health awareness, but 50% of them reported visiting a dental clinic, and only 26% of them reported tooth brushing twice daily. In the DM group, 63% of the parents reported oral-health awareness, 88% of their children had visited a dentist, and only 12% reported brushing teeth twice daily. Although 90% of the parents in the ACT group reported awareness to the oral-health aspects of their children's disease, only 60% of them reported that their children visited dentists, and only 38% reported that their children brushed their teeth twice daily. These gaps between awareness and behavior highlight that physicians specializing in particular diseases should inform parents about the association and the importance of maintaining oral health, and also refer them to dentists as part of the children's periodic follow-ups. The dentists are responsible for providing the specific instructions regarding dental and oral health. After thorough knowledge of a child's medical condition is gained and any special care requirements are identified, a definitive dental treatment plan can be established. Preventive dentistry including advice on diet and oral hygiene, and home and office fluoride therapy, should be provided for all pediatric patients. Similar cooperation with other pediatric departments can promote parental awareness and improve children's dental hygiene habits.

The study has several limitations. First is the possibility of response bias, as the respondents may have desired to answer questions in a way they believed would meet the interviewers’ expectations. Second, some of the results might be influenced by the cooperation between the Pediatric Dentistry Department and the Hematology-Oncology Department, and might not represent attitudes of parents of children treated at other medical centers. Moreover, a reporting bias should be considered, as the dental habits and dental visits presented are according to parental reports. We focused on 3 groups of systemic, life-threatening diseases in children, but there are other chronic diseases and systemic conditions that were not included for example genetic syndromes, nephrological patients, children with cerebral palsy and more, which are worth examining in the following studies. Another limitation is the size of the control group, the healthy children, which included 61 questionnaires out of the 287 questionnaires in the study.

## Data Availability

The raw data supporting the conclusions of this article will be made available by the authors, without undue reservation.
